# Far lateral lumbar intervertebral disc extrusion in a cat: Case report

**DOI:** 10.17221/107/2024-VETMED

**Published:** 2025-09-30

**Authors:** Youjung Jang, Hyung-Kyu Chae, Yeon-Jung Hong

**Affiliations:** ^1^Department of Veterinary Radiology, Western Referral Animal Medical Center, Seoul, Republic of Korea; ^2^Department of Veterinary Radiology, My Referral Animal Medical Center, Paju, Republic of Korea; ^3^Department of Veterinary Clinical Nutrition, Kyungpook National University, Daegu, Republic of Korea; ^4^Korea Society of Feline Medicine, Seoul, Republic of Korea; ^5^Department of Veterinary Surgery, Western Referral Animal Medical Center, Seoul, Republic of Korea; Youjung Jang and Hyung-Kyu Chae contributed equally to this work

**Keywords:** feline, herniation, lameness, magnetic resonance imaging, spinal disorder

## Abstract

A 4-year-old domestic long-haired cat presented with acute, progressive, non-weight-bearing lameness of the left pelvic limb and reluctance to climb. Magnetic resonance imaging (MRI) revealed decreased T2-weighted signal intensity of the nucleus pulposus in all lumbar intervertebral discs except L7-S1, with far-lateral T2-weighted low-signal material at L6–L7 surrounding the sixth lumbar nerve root. A diagnosis of far-lateral intervertebral disc extrusion at L6–L7 was made. Clinical signs resolved almost completely within 15 days of conservative management. To our knowledge, this is the first report describing MRI findings of far-lateral intervertebral disc extrusion in a cat, highlighting the importance of considering this condition in the differential diagnosis of acute unilateral pelvic limb lameness.

Intervertebral disc disease (IVDD) is uncommon in cats, accounting for approximately 5% of all spinal disorders and occurring less frequently than in dogs (Marioni-Henry et al. 2004; Goncalves et al. 2009; Hamilton-Bennett and Behr 2019). The most commonly reported types include acute non-compressive nucleus pulposus extrusion (ANNPE), intervertebral disc protrusion (IVDP), and intervertebral disc extrusion (IVDE), which most frequently affect the thoracolumbar and lumbosacral regions (Hamilton-Bennett and Behr 2019; Debreuque et al. 2020).

Atypical herniations, such as foraminal or far-lateral lumbar disc extrusions, have been reported in dogs and often present as unilateral lameness, mimicking orthopaedic disorders (Fadda et al. 2013; Silva et al. 2022). In humans, these lesions are frequently managed surgically due to the severity of the pain. In dogs, however, treatment outcomes vary and may depend on the severity of clinical signs. However, to our knowledge, only one case of lumbar foraminal disc extrusion has been documented in cats (Ryan and Cherubini 2022).

To our knowledge, this is the first report to describe a far-lateral intervertebral disc extrusion at L6–L7 in a cat, as diagnosed using magnetic resonance imaging (MRI).

## Case description

A 4-year-old neutered female domestic long-haired cat was referred to the Western Referral Animal Medical Centre with a chief complaint of acute, progressive, non-weight-bearing lameness in the left pelvic limb and reluctance to climb structures such as cat towers.

**Figure 1:**
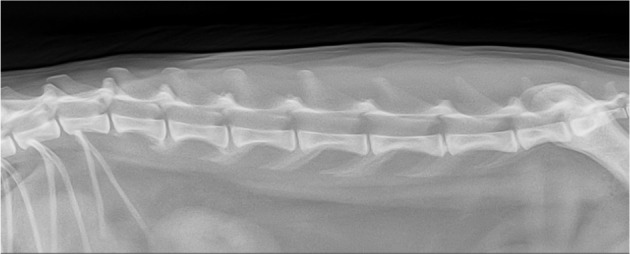
Right lateral radiographic image of the lumbar spine showing no significant abnormalities

Owing to patient defensiveness, physical and neurological examinations were not feasible. However, echocardiography and blood tests, including glucose and lactate levels, showed no evidence of cardiovascular diseases such as aortic thromboembolism. Radiography of the lumbar spine and pelvic limbs revealed no remarkable findings (Figure 1).

Since the owner declined further diagnostics at that time, the cat was discharged with meloxicam [0.05 mg/kg p.o. q24h (Metacam; Boehringer Ingelheim Vetmedica, St. Joseph, MO, USA)] and gabapentin [11 mg/kg (Gabapentin cap.; Donga Pharm, Seoul, Republic of Korea)] for 7 days. Despite treatment, the symptoms persisted, and the lameness continued to progress, prompting an MRI evaluation. Pre-anaesthetic haematological and biochemical tests showed no significant abnormalities.

MRI was performed using a 1.5 Tesla high-field scanner (Achieva; Philips Healthcare, Best, Netherlands) to evaluate the lumbosacral spinal cord and associated structures. The protocol included sagittal T2-weighted short tau inversion recovery (STIR) and transverse T2- and T1-weighted sequences, with and without fat-saturated post-contrast imaging.

**Figure 2:**
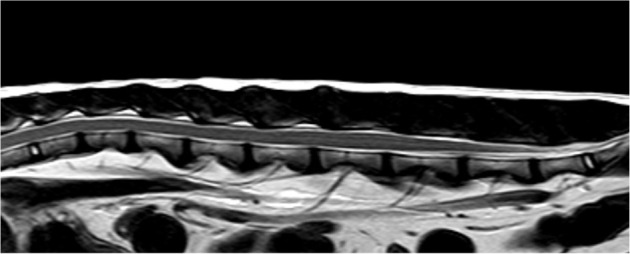
Sagittal T2-weighted MRI of the lumbar spine showing loss of normal hyperintense signal in the nucleus pulposus from T12 to L7, consistent with disc degeneration

**Figure 3:**
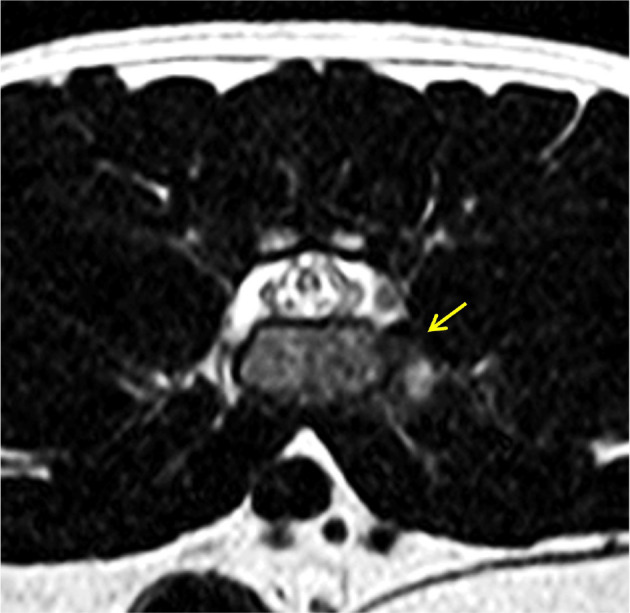
Transverse T2-weighted image at L6–L7 showing left-sided hypointense disc material (yellow arrow) outside the intervertebral foramen, adjacent to the vertebral body and transverse process, compressing the nerve root, with focal hyperintensity in the adjacent epaxial muscles

**Figure 4:**
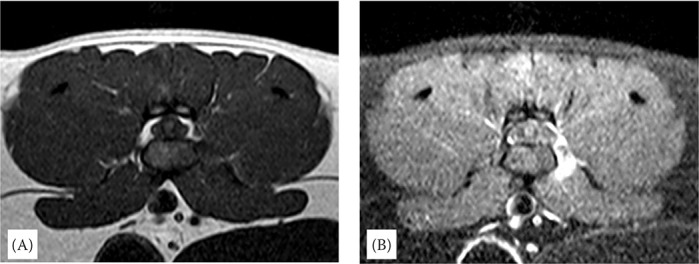
(A) T1-weighted image showing left far lateral disc herniation. (B) Postcontrast transverse image demonstrating perilesional contrast enhancement and nerve root involvement, with hypointense disc material surrounded by enhancing inflammatory tissue

On sagittal T2-weighted and STIR sequences, the nucleus pulposus of all lumbar discs (except L7–S1) showed decreased signal intensity, consistent with disc degeneration (Figure 2). At L6–L7, the left-sided hypointense disc material impinged on the sixth lumbar nerve root. Hyperintensity in the adjacent left epaxial musculature was also observed (Figure 3). Post-contrast images showed marked perilesional enhancement adjacent to the herniated disc material (Figure 4A,B). These findings supported the diagnosis of left-sided far-lateral disc extrusion at L6–L7 with associated neuritis.

Surgical intervention was considered if the medical treatment failed. The patient was prescribed prednisolone [0.5 mg/kg p.o. q12h (Sorondo; Yuhan Co. Ltd., Seoul, Republic of Korea)] for 7 days. Symptoms improved within 5 days, with resolution of lameness by day 15 post-onset. Prednisolone was tapered to 0.3 mg/kg over one week, then discontinued over the following two weeks. At the 2-month follow-up, the cat remained symptom-free. To date, no surgical treatment has been reported for cats, and further research is needed to determine the necessity of a surgical approach to remove herniated soft disc material.

## DISCUSSION AND CONCLUSION

Intervertebral disc extrusion most commonly affects chondrodystrophic dogs and typically begins early in life (Mai 2018). It is rare in cats and primarily affects certain purebred breeds, such as British Shorthair and Persian cats (De Decker et al. 2017). Cranial thoracic extrusions (above T10) are uncommon because of the presence of the intercapital ligaments. In cats, the L4–S3 and T3–L3 regions are most frequently affected (Mai 2018).

Atypical degenerative disc extrusion, particularly foraminal or far-lateral extrusion, typically causes nerve root compression rather than spinal cord compression. According to Mai (2018), this condition may be more common in the cervical segment because of the relatively thick annulus in that region. The clinical signs include pain and limb elevation in a non-weight-bearing posture, indicating radiculopathy (Schachar et al. 2024). Although cervical foraminal extrusions are being increasingly recognised in dogs, thoracolumbar far-lateral extrusions remain rare (Fadda et al. 2013; Silva et al. 2022).

In a study of 37 dogs, 87% of foraminal or far-lateral thoracolumbar herniations occurred at L5–L6 or L6–L7. Of the 38 herniated discs, 16 involved both the foraminal and far-lateral areas, whilst 13 were exclusively far-lateral (Silva et al. 2022).

In the present case, far-lateral disc extrusion was diagnosed using MRI. Although a full neurological assessment was limited, the observed acute-onset progressive unilateral pelvic limb lameness was consistent with similar cases in dogs (Fadda et al. 2013).

In contrast to a previously reported feline case of lumbar intervertebral foraminal disc extrusion (Ryan and Cherubini, 2022), in which the disc material protruded into the intervertebral foramen and caused ipsilateral pelvic limb lameness, our case demonstrated a more laterally displaced far-lateral extrusion. Furthermore, prominent MRI changes were observed in the adjacent epaxial musculature, suggesting an extensive local inflammatory or reactive process.

In this case, NSAID treatment was ineffective after one week, consistent with canine cases requiring surgery (Fadda et al. 2013). However, the symptoms resolved after corticosteroid therapy. Conservative treatments, including meloxicam and gabapentin, have shown favourable outcomes in some canine and feline cases (Ryan and Cherubini 2022; Silva et al. 2022).

An extruded intervertebral disc may appear as mineralised material on lateral radiographs when a calcified disc extrudes into the vertebral canal or the intervertebral foramen above it.

Radiographs of this patient did not reveal any extruded mineralised discs of the lumbar vertebral column, unlike previous reports of foraminal disc extrusion observed in dogs and cats. Similarly, low-signal disc material extruding into the spinal canal or foramen was not observed in the sagittal plane on MRI (Ryan and Cherubini 2022; Silva et al. 2022). Therefore, careful attention to transverse plane images is required for the diagnosis of far lateral disc extrusion, with due consideration of the presence or absence of neuritis. Even in the absence of a narrow disc space or disc material protruding into the vertebral canal or foramen on sagittal plane images, a disc that extrudes far laterally must be identified on transverse plane images, along with the presence or absence of neuritis on MRI to confirm the diagnosis. Furthermore, in cases of inflammation around the extruded disc material, unilateral hyperintensity of the epaxial musculature adjacent to the herniated disc material was identified on STIR images, revealing strong enhancement of the perilesional soft tissue on T1-weighted contrast-enhanced images, which is useful for detecting disc extrusion and inflammatory changes. Despite limitations in the neurological examination and the inability to perform follow-up MRI, the patient’s complete clinical recovery suggests a favourable prognosis. Additional case reports on far-lateral disc extrusion in cats and the associated physical examination findings are needed.

In conclusion, this report described the imaging features of far-lateral disc extrusion in cats. In cases of acute onset of unilateral pelvic limb lameness in cats, which is particularly challenging for neurological and physical examinations compared to dogs, far lateral disc herniation must be considered as a differential diagnosis based on MRI findings.
